# Association Between Sarcopenia Risk and Dysphagia Risk in Older Adults Not Certified for Long-Term Care Insurance in Japan

**DOI:** 10.7759/cureus.89805

**Published:** 2025-08-11

**Authors:** Mayumi Nasu, Hiroshi Kishimoto, Satoko Yano, Yumi Chiba, Kazuhiro Ichimura, Ikuko Nakamura, Kumiko Ito, Chikako Kodama, Kumiko Ichimura

**Affiliations:** 1 Department of Nursing, Kameda College of Health Sciences, Kamogawa, JPN; 2 Department of Nursing, Faculty of Health Sciences, Ibaraki Prefectural University of Health Sciences, Ami, JPN; 3 Department of Rehabilitation Medicine, Ibaraki Prefectural University of Health Sciences, Ami, JPN; 4 Department of Nursing, Graduate School of Medicine, Yokohama City University, Yokohama, JPN; 5 Department of Oral Medicine, Ichimura Dental Clinic, Tsuchiura, JPN; 6 Department of Oral Medicine, Ibaraki Dental Hygienists’ Association, Mito, JPN; 7 Department of Clinical Nutrition, The Ibaraki Dietetic Association, Mito, JPN; 8 Department of Nutrition, Ibaraki Prefectural University of Health Sciences, Ami, JPN

**Keywords:** dysphagia risk, eat-10, sarc-f, sarcopenia risk, sarcopenic dysphagia

## Abstract

Aim: This study aimed to investigate the risk of sarcopenia and dysphagia in community-dwelling older adults aged 75 years and older who are not certified for long-term care insurance.

Methods: This cross-sectional study used self-administered questionnaires, including the SARC-F for sarcopenia risk and the Eating Assessment Tool-10 (EAT-10) for dysphagia risk, mailed to 1,000 randomly selected community-dwelling older adults aged 75 years and older who were not certified for long-term care insurance in Japan. Participants were classified according to their risk of sarcopenia (SARC-F ≥4) or dysphagia (EAT-10 ≥3). The association between sarcopenia and dysphagia risks was analyzed using chi-squared tests and logistic regression analysis, adjusting for age, sex, body mass index, and previous history of diseases that may cause dysphagia.

Results: Of 568 participants (median age 79.0 years, 282 {49.6%} male) with valid responses, 68 (12.0%) and 93 (16.4%) were in the sarcopenia and dysphagia risk groups, respectively. A significantly higher proportion of participants in the sarcopenia risk group were also classified in the dysphagia risk group (32 {47%} participants, p<0.001). Logistic regression analysis confirmed the association between sarcopenia risk and dysphagia risk (odds ratio: 5.2; 95% confidence interval: 2.91-9.29).

Conclusions: We identified an association between sarcopenia and dysphagia risk in community-dwelling older adults who are not certified for long-term care insurance, with a significantly higher proportion of the sarcopenia risk group at risk for dysphagia. Our findings suggest that incorporating dysphagia screening into existing community-based sarcopenia screening efforts may be an effective approach to identifying older adults at risk for both conditions.

## Introduction

Dysphagia is widely known to negatively impact the life expectancy and quality of life of older adults. Impaired swallowing ability is a major risk factor for aspiration pneumonia in this population [1]. Previous studies have reported that dysphagia is associated with malnutrition [2], which is a risk factor for secondary sarcopenia [3]. Other risk factors include physical inactivity and diseases, such as inflammatory diseases and malignancies [3]. The European Working Group on Sarcopenia in Older People (EWGSOP2) stated in 2019 that sarcopenia is a muscle disease rooted in adverse muscle changes that accrue over a lifetime [4]. The prevalence of sarcopenia in community-dwelling older adults is reported to be approximately 15% [5].

Previous studies have shown that sarcopenia increases the risk of physical disability, falls, hospitalization, and death [6]. Recent reports have shown that it leads to a decrease in muscle mass and strength of muscles involved in swallowing, possibly resulting in decreased swallowing function [7]. These previous studies have established the concept of “sarcopenic dysphagia” [8]. Therefore, it is important to understand the current situation of sarcopenic dysphagia from a prevention perspective [8]. Furthermore, because sarcopenic patients are likely to be unaware of their own common symptoms, appropriate diagnosis and intervention are rarely performed [9]. The same situation applies to patients with dysphagia, and it has been reported that many patients with dysphagia are unaware of their own dysphagia [10]. As a result, people with potential sarcopenia, dysphagia, or both, who have no subjective symptoms, may become severely ill without warning and fall into a downward spiral of dysphagia and sarcopenia. Early screening should, therefore, be used to identify high-risk patients and provide early interventions. Therefore, screening people with subclinical sarcopenia, dysphagia, or both, who are unaware of their symptoms, is necessary. Self-administered questionnaires are useful and widely implemented to screen community-dwelling individuals at risk of potential health problems in various specialties, such as chronic diseases, nutrition, and geriatrics [11-13]. Valid and reliable self-administered questionnaires used for such screening include the SARC-F for sarcopenia [14,15] and the Eating Assessment Tool-10 (EAT-10) for dysphagia (table and figure in appendix) [16,17].

No studies have examined the risk of sarcopenia and dysphagia and their association in community-dwelling older adults who are not dependent on the long-term care insurance (LTCI) system. The LTCI system was introduced in Japan in the year 2000 as a public social insurance system. Japanese older adults who have been certified as needing LTCI services can receive institutional, in-home, and community-based services depending on their physical and cognitive disabilities [18]. A previous study assessed the association between swallowing function, nutritional status, and physical function in community-dwelling older adults with disabilities who received LTCI and used daycare services [19]. Another study evaluated the association between dysphagia and sarcopenia in patients aged 60 years and older who attend a geriatric outpatient clinic [20]. These studies used face-to-face surveys for data collection [19,20]. In the former [19], an association was found between swallowing function scores assessed by the Seirei Dysphagia Screening questionnaire [21] and SARC-F scores [14,15]. In the latter [20], an association was found between swallowing function scores assessed by the EAT-10 [16,17] and SARC-F scores [14,15].

This study aimed to examine whether a relationship exists between sarcopenia and dysphagia risk among community-dwelling Japanese adults aged 75 years and older who are not certified for the LTCI system. Using mailed, self-administered questionnaires, we sought to identify individuals with potential subclinical symptoms of both conditions, which might facilitate early screening and intervention strategies in this relatively independent older population.

## Materials and methods

Study design

This study employed a cross-sectional design, conducted via mail, and utilized anonymous self-administered questionnaires. It was conducted as a collaborative project between Ibaraki Prefectural University of Health Sciences and a local municipality (Ami Town, Ibaraki Prefecture, Japan).

Participants

In November 2022, 1,000 local residents aged 75 years or older living in Ami town who were not LTCI-certified were considered for this study. The self-administered questionnaires were mailed to 521 (52.1%) persons aged 75-79 years (236 males and 285 females), 307 (30.7%) persons aged 80-85 years (146 males and 161 females), 130 persons aged 85-89 years (57 males and 73 females), and 42 (4.2%) persons aged 90 years and older (18 males and 24 females) who were randomly selected.

Assessment of sarcopenia risk

The Japanese version of the SARC-F scale is used to assess the risk of sarcopenia [22]. The SARC-F scale was validated for internal consistency, as well as for factorial, criterion-related, and construct validity [15]. The European Working Group on Sarcopenia in Older People (EWGSOP2) [4] and Asian Working Group for Sarcopenia (AWGS) have also recommended its use as a screening tool for sarcopenia [23]. The Japanese version of the SARC-F scale was developed by Tanaka et al. and Ida et al. after forward translation of the original scale, backward translation, and author verification [22,24]. The Japanese version has also been tested for reliability and validity in patients with cardiac diseases [22] and diabetes [24]. The questions were based on the following five items: strength (S; weakness), assistance with walking (A; with or without walking aids), rising from a chair (R; getting up from a chair), climbing stairs (C; climbing stairs), and falls (F; falling down). Each item was rated on a scale of 0 to 2, with 0 being “not difficult at all” and 2 being “very difficult.” The total score ranged from 0 to 10. The cut-off value was 4 points or higher.

Assessment of dysphagia risk

The Japanese version of the EAT-10, translated by Wakabayashi and Kayashita, was used to assess dysphagia risk [25]. The EAT-10 has been validated for internal consistency, test repeatability, and criterion-relevant validity [15,16]. The forward and backward translation processes were used to validate internal consistency and criterion-relevant validity of EAT-10 [24]. The questionnaire consisted of 10 questions, each scored from 0 (no problem) to 4 (severely problematic), and the total score was calculated. The cut-off value was three points or higher.

Other questions

The participants were asked to fill in their age, sex, height, weight, smoking status, current medical history, previous medical history, and regular medications. The participants were also asked whether they had a history of stroke, neuromuscular disease, or cancer of the oral cavity or throat, each of which could cause dysphagia.

Statistical analysis

IBM SPSS Statistics version 28 (Armonk, NY: IBM Corp.) was used for the statistical analysis. The Shapiro-Wilk test was used to assess the normality of the data. Normally distributed data are expressed as mean and standard deviation (SD); non-normally distributed data are expressed as median and interquartile range (IQR); Student’s t-test and Mann-Whitney U test were used to analyze differences between categories, as appropriate. Categorical data are expressed as frequencies and percentages, and chi-squared tests were used for comparisons, as appropriate. Logistic regression analysis was performed with the risk of dysphagia (cut-off value: 3 points) as the objective variable and risk of sarcopenia (cut-off value: 4 points), age, sex, BMI, and previous history of diseases that can cause dysphagia (stroke, neuromuscular disease, and oral cavity or throat cancer) as the explanatory variables. A p<0.05 was considered statistically significant.

Ethics

This study was approved by the Ethics Review Committee of Ibaraki Prefectural University of Health Sciences (approval no. 1057). All study participants filled out the research consent form along with the questionnaire when they received them via email. A valid response indicated the return of a signed consent form and a filled questionnaire.

## Results

**Figure 1:**
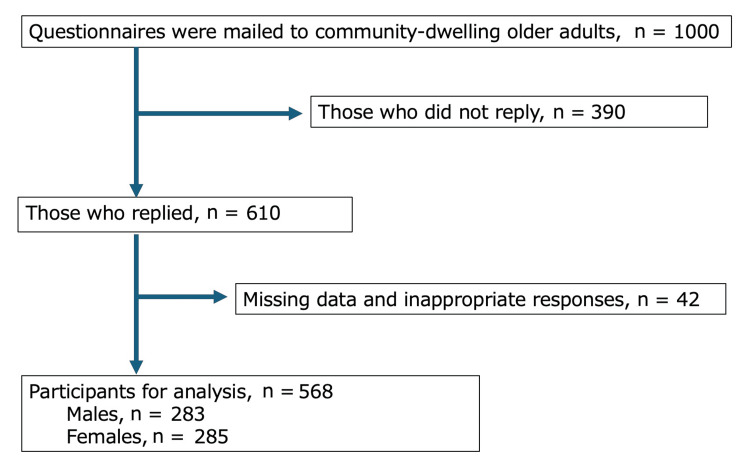
Flowchart of participants.

Of the 1,000 people who received the questionnaires, 610 (61.0%) responded, of whom 568 (56.8%, median age 79.0 years; 49.6% male) were eligible for analysis. We excluded 42 people with missing data or inappropriate responses, specifically those with incomplete responses that prevented proper scoring of either the SARC-F or EAT-10 questionnaires, and cases where key variables required for logistic regression analysis were missing (age, sex, BMI, and previous history of diseases that may cause dysphagia) (Figure 1).

The demographic and clinical characteristics of the study population are shown in Table 1. The overall median age was 79 years with an interquartile range of 76-82 years. Only 18 participants (3.2%) reported a history of conditions that could potentially cause dysphagia, including stroke, neuromuscular disease, or oral or pharyngeal cancer.

**Table 1:** Baseline characteristics of participants by dysphagia risk status. ^a^Chi-squared test. ^b^Mann-Whitney U test. EAT-10: Eating Assessment Tool-10

Variables	Overall	EAT-10 <3	EAT-10 ≥3	Test statistics	p-Value
		(n=475, 83.6%)	(n=93, 16.4%)		
Men (%)	283 (49.8)	235 (83.0)	48 (17.0)	0.142	0.706^a^
Women (%)	285 (50.2)	240 (84.2)	45 (15.8)	-	-
Age, median (IQR)	79 (76-82)	79 (76-81)	81 (78-85)	28,935	<0.001^b^
Body mass index, median (IQR)	22 (21-24.25)	22 (21-25)	22 (20-24)	19,141	0.041^b^
Previous history of diseases that may cause dysphagia (+) (%)	18 (3.2)	14 (77.8)	4 (22.2)	0.464	0.496^a^
Previous history of diseases that may cause dysphagia (-) (%)	550 (96.8)	461 (83.8)	89 (16.2)	-	-
SARC-F <4 (%)	500 (88.0)	439 (87.8)	61 (12.2)	53.1	<0.001^a^
SARC-F ≥4 (%)	68 (12.0)	36 (52.9)	32 (47.1)	-	-
SARC-F, median (IQR)	0 (0-2)	0 (0-1)	2 (0-4)	30,817	<0.001^b^
EAT-10, median (IQR)	0 (0-1)	0 (0-0)	8 (4-10)	44,175	<0.001^b^

Using the SARC-F sarcopenia risk assessment questionnaire, 68 participants (12.0%) scored 4 or more points and were classified as being at risk for sarcopenia. The median SARC-F score for the entire cohort was 0 points with an interquartile range of 0-2 points, indicating that most participants had minimal functional limitations.

Using the EAT-10 assessment tool to evaluate the risk of dysphagia, 93 participants (16.4%) scored 3 points or higher and were classified as being at risk for dysphagia. The median score was 0, with a quartile range of 0-1, indicating that the majority of participants did not have symptoms of dysphagia.

The key finding was the significant overlap between these two risk categories. Among the 68 participants at risk for sarcopenia, 32 people (47.1%) were also at risk for dysphagia, compared to only 61 people (12.2%) at risk for dysphagia among the 500 people not at risk for sarcopenia. This difference was statistically significant, showing a strong association between the two conditions. Participants in the dysphagia risk group were significantly older, with a median age of 81 years compared to 79 years in the non-risk group. No significant difference was observed between the sexes with regard to dysphagia risk.

Logistic regression analysis confirmed the independent association between sarcopenia risk and dysphagia risk after controlling for potential confounders (Table 2). Age emerged as a significant predictor, with each additional year associated with increased odds of dysphagia risk. Participants at risk of sarcopenia had an odds ratio of 5.17 (95% CI: 2.89-9.22) for dysphagia risk compared to those without sarcopenia risk. Of the 32 participants at risk for both conditions, only one reported a history of stroke, neuromuscular disease, or oral/pharyngeal cancer.

**Table 2:** Logistic regression analysis with dysphagia risk as the outcome.

Variables	B	Odds ratio	95% Confidence interval of odds ratio		Wald statistic	p-Value
			Lower	Upper		
Sex	-0.222	0.801	0.494	1.298	0.811	0.368
Age	0.081	1.084	1.026	1.145	8.378	0.004
Body mass index	-0.078	0.925	0.851	1.005	3.434	0.064
Previous history of diseases that may cause dysphagia	0.419	1.521	0.646	3.581	0.922	0.337
SARC-F ≥4	1.643	5.17	2.889	9.218	31.001	<0.001

## Discussion

It was found that 12.0% (68) of community-dwelling older adults not certified for LTCI were at risk for sarcopenia and 16.4% were at risk for dysphagia. In a study using the SARC-F to assess the prevalence of sarcopenia in community-dwelling older adults, 15.4% of participants were found to have sarcopenia [15]. In another study using the EAT-10 to assess the prevalence of dysphagia, 25.1% of participants were found to have dysphagia [26]. The results of this study showed that despite the median age of the oldest participants, the proportion of people at risk of sarcopenia and dysphagia was lower than the prevalence rates mentioned above. This difference may be due to the fact that the participants in this study, although older, were not dependent on the LTCI system and were in better health than participants in other studies.

The statistically significantly higher proportion of the group at risk of sarcopenia in this study was also at risk of dysphagia (32 out of 68, 47.1%), suggesting an association between the risk of sarcopenia and the risk of dysphagia in community-dwelling older adults. This association was also observed after adjusting for age, sex, body mass index, and history of diseases that may cause dysphagia using logistic regression analysis. These results suggest that identifying older adults at risk of sarcopenia may increase the likelihood of identifying older adults at risk of dysphagia. Currently, many local governments and communities are conducting research and implementing interventions related to sarcopenia by multiple researchers [27,28]. Therefore, incorporating an approach to dysphagia into these efforts may allow for more efficient support of potential community-dwelling older adults at risk of dysphagia.

The combined sarcopenia and dysphagia risk population in this study could be screened for potential patients with and at risk of sarcopenic dysphagia. Sarcopenic dysphagia is diagnosed using diagnostic algorithms that are reliable and valid, and the criteria include both systemic sarcopenia and dysphagia [29]. The algorithm excludes cases with neuromuscular disease or other causes of dysphagia other than sarcopenia. Of the 32 (5.6%) patients in this study who had both sarcopenia and dysphagia risks, only one patient indicated current or previous history of a disease that could cause dysphagia. In conjunction with the diagnostic algorithm for sarcopenic dysphagia, 31 patients could qualify as patients with sarcopenic dysphagia [29]. The updated EWGSOP2 recommendations aim to increase awareness of sarcopenia and its risks, and call on healthcare professionals who treat patients at risk of sarcopenia to develop strategies for early detection and treatment [4]. Furthermore, the AWGS highlights the impact of sarcopenia in all healthcare settings and recommends individualized lifestyle interventions ﻿that can be implemented across various healthcare fields [23]. Hospital-based studies of sarcopenic dysphagia have reported poorer improvement in activities of daily living and swallowing function compared with that observed in other dysphagia cases [30]. Thus, screening for potential patients with and at risk of sarcopenic dysphagia may be useful for the early improvement of dysphagia.

This study has several limitations. First, it has been suggested that an individual who is unable to respond to the EAT-10 is likely to have mild or low-grade dysphagia [25]. It is possible that some of the residents who did not return the questionnaire in this study were unable to answer the questions. This suggests that the questionnaire in this study may not have been able to adequately screen for people at risk of dysphagia. Second, because the screening survey was conducted by mail, sarcopenia and dysphagia were not directly diagnosed. In the future, surveys that include direct diagnosis should be planned to verify the reliability and validity of self-administered questionnaires. Third, the cross-sectional design of this study is a significant limitation that must be emphasized. While we demonstrated a statistically significant association between sarcopenia risk and dysphagia risk, we cannot establish causality or determine the temporal relationship between these conditions. It remains unclear whether sarcopenia leads to dysphagia, dysphagia contributes to sarcopenia development, or both conditions arise from common underlying factors, such as malnutrition, aging-related physiological changes, or other shared pathophysiological mechanisms. This limitation is particularly important when interpreting the clinical implications of our findings, as the direction of causality would influence intervention strategies. Future longitudinal studies are needed to clarify the temporal relationship and causal pathways between sarcopenia and dysphagia in community-dwelling older adults.

## Conclusions

This study revealed a significant association between the risk of sarcopenia and the risk of dysphagia in community-dwelling older adults aged 75 years or older who were not certified for long-term care insurance in Japan. Our results show that 47.1% of those at risk for sarcopenia were also at risk for dysphagia, with an odds ratio of 5.17 compared to those without sarcopenia risk. This association remained statistically significant after adjustment for age, sex, body mass index, and history of conditions that may cause dysphagia. Based on these findings, incorporating dysphagia screening into existing community-based sarcopenia screening programs may be a useful approach to identify older adults at risk of dysphagia. This integrated screening strategy could help to identify individuals with subclinical symptoms who could benefit from early intervention. Further research is needed to validate the effectiveness of such combined screening approaches and to determine optimal intervention strategies for individuals identified as at risk for both sarcopenia and dysphagia in community settings.

## Figures and Tables

**Figure 2:**
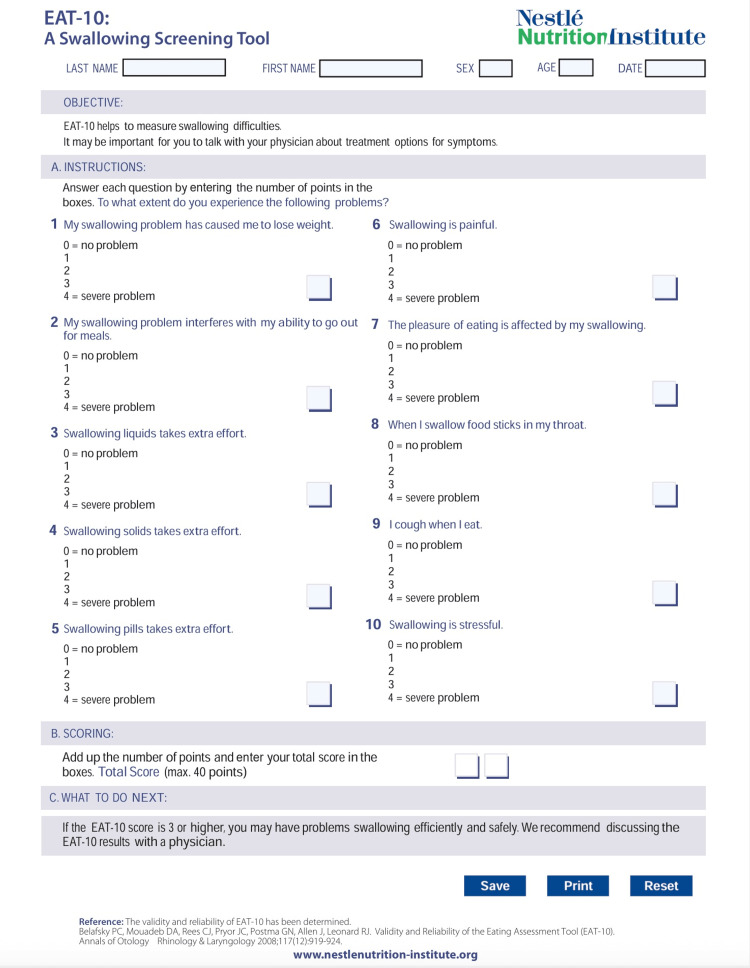
The Eating Assessment Tool-10 (EAT-10) (original). Permission to use the original version of EAT-10 [16,17] and the Japanese version [25] was obtained from MAPI Research Trust.

## Appendices

**Table 3:** SARC-F screen for sarcopenia (original). Permission to use the original version of SARC-F [14] (from the publisher) and the Japanese version [22] (from the author) was obtained from the respective copyright holders.

Component	Question	Scoring
Strength	How much difficulty do you have in lifting and carrying 10 pounds?	None = 0, some = 1, a lot or unable = 2
Assistance in walking	How much difficulty do you have walking across a room?	None = 0, some = 1, a lot, use aids, or unable = 2
Rise from a chair	How much difficulty do you have transferring from a chair or bed?	None = 0, some = 1, a lot or unable without help = 2
Climb stairs	How much difficulty do you have climbing a flight of 10 stairs?	None = 0, some = 1, a lot or unable = 2
Falls	How many times have you fallen in the past year?	None = 0, 1-3 falls = 1, 4 or more falls = 2
